# Systematic Review of Micro-RNA Expression in Pre-Eclampsia Identifies a Number of Common Pathways Associated with the Disease

**DOI:** 10.1371/journal.pone.0160808

**Published:** 2016-08-16

**Authors:** Adam M. Sheikh, Heather Yvonne Small, Gemma Currie, Christian Delles

**Affiliations:** Institute of Cardiovascular and Medical Sciences, College of Medical, Veterinary and Life Sciences, University of Glasgow, Glasgow, United Kingdom; Universidade de Sao Paulo Instituto de Ciencias Biomedicas, BRAZIL

## Abstract

**Background:**

Pre-eclampsia (PE) is a complex, multi-systemic condition of pregnancy which greatly impacts maternal and perinatal morbidity and mortality. MicroRNAs (miRs) are differentially expressed in PE and may be important in helping to understand the condition and its pathogenesis.

**Methods:**

Case-control studies investigating expression of miRs in PE were collected through a systematic literature search. Data was extracted and compared from 58 studies to identify the most promising miRs associated with PE pathogenesis and identify areas of methodology which could account for often conflicting results.

**Results:**

Some of the most frequently differentially expressed miRs in PE include miR-210, miR-223 and miR-126/126* which associate strongly with the etiological domains of hypoxia, immunology and angiogenesis. Members of the miR-515 family belonging to the imprinted chromosome 19 miR cluster with putative roles in trophoblast invasion were also found to be differentially expressed. Certain miRs appear to associate with more severe forms of PE such as miR-210 and the immune-related miR-181a and miR-15 families. Patterns of miR expression may help pinpoint key pathways (e.g. IL-6/miR-223/STAT3) and aid in untangling the heterogeneous nature of PE. The detectable presence of many PE-associated miRs in antenatal circulatory samples suggests their usefulness as predictive biomarkers. Further progress in ascertaining the clinical value of miRs and in understanding how they might contribute to pathogenesis is predicated upon resolving current methodological challenges in studies. These include differences in diagnostic criteria, cohort characteristics, sampling technique, RNA isolation and platform-dependent variation in miR profiling.

**Conclusion:**

Reviewing studies of PE-associated miRs has revealed their potential as informants of underlying target genes and pathways relating to PE pathogenesis. However, the incongruity in results across current studies hampers their capacity to be useful biomarkers of the condition.

## Introduction

Few conditions which affect pregnancies are as prevalent, threatening and as steeped in theory as pre-eclampsia (PE)—a multi-factorial, systemic and almost entirely human condition. PE remains one of the primary drivers of maternal and perinatal morbidity and mortality throughout the world, besetting up to 8% of all pregnancies, causing 10% of maternal deaths and responsible for nearly 5% of stillbirths in the UK [1,2]. Typically occurring during the 3^rd^ trimester, this condition bears with it characteristic symptoms which include hypertension and proteinuria [3]. Though primarily regarded as a hypertensive condition, the dangers of PE extend beyond, into a multitude of systemic effects such as renal failure, pancreatitis and haemolytic anaemia [4]. Symptoms often surface in normotensive, non-proteinuric women and emerge in grades of severity, which can progress towards the grand-mal seizures of eclampsia and ultimately systemic maternal organ damage or death [5].

Proposed aetiological domains for PE span the fields of genetics, immunology and endocrinology. Implicated risk factors include maternal age, nulliparity and the presence of pre-existing conditions such as diabetes or hypertension [6]. Ethnicity, including mixed ethnicities, may also be a contributing factor [7]. These risk factors are entwined with socioeconomic factors, where individuals from lower socioeconomic backgrounds are at a heightened risk of PE [8]. The breadth of risk and adverse outcomes highlights the importance of early diagnosis—however, a consistently agreed-upon definition and classification of PE remains a contentious issue. PE is generally classified into mild (mPE) and severe (sPE) using thresholds parameters for systolic blood pressure, diastolic blood pressure and proteinuria; typically in accordance with ISSHP criteria [9]. This is in contrast with temporal classification into early- (eoPE) and late-onset PE (loPE), usually assigned in accordance with the presentation of PE features at gestational age (GA) thresholds of either <34 weeks or >34 weeks respectively [10].

As PE pathology can be progressive, classification into mild and severe may not accurately depict the risk associated with the opaque and transient nature of the condition. For example, around 10% of women have no presence of hypertension or proteinuria within a week of their initial eclamptic episode [11]. In lieu of this, the presence of severe features alongside baseline PE symptoms has been suggested [12]. PE is also frequently co-morbid with other conditions such as intra-uterine growth restriction (IUGR) or small-for-gestational age (SGA), and symptoms can often “mimic”, or overlap, with those of other syndromes such as HELPP syndrome—making diagnosis a distinctly non-trivial matter [13]. Coupled with clinical pressures surrounding the risk of under-diagnosis, this has resulted in a dilution of diagnostic specificity for PE [14]. There is, therefore, a need to clarify the underlying pathogenic mechanisms of PE, in order to unambiguously distinguish the condition and open the doors to early prognostic and therapeutic measures.

The central hypothesis for understanding PE pathogenesis consists of two stages: starting with abnormal placentation and uteroplacental ischemia early in pregnancy and progressing towards a maternal inflammatory response [5]. In normal pregnancy, invasion of the decidual lining by fetally-derived extravillous trophoblasts surrounding the incipient blastocyst is a crucial step in remodelling the maternal spiral arteries to adequately perfuse the developing placenta and fetus [15]. Interstitial trophoblasts invade the myometrium layer of the uterine wall, releasing vasodilatory factors such as nitric oxide, preparing the spiral arteries ahead of invasion, attachment, destruction and replacement of the arterial lining by endovascular cytotrophoblasts [14]. In PE, inadequate remodelling of the spiral arteries by these invasive trophoblasts is thought to result in under-perfusion of the placenta.

A number of theories have been proposed to account for defective arterial remodelling at this stage. Firstly, endovascular trophoblasts with particular haplotypes of the cell-surface human leukocyte antigen may interact with natural killer cells and T-cells of the maternal immune system in ways that could affect normal maternal-foetal immune adaptation [16,17]. Secondly, improper preconditioning of the uterus through menstruation has been proposed to be a cause [18].

Thirdly, as invasion is predicated upon an increase in oxygen tension associated with a rise in blood flow in early pregnancy; it is possible that impaired invasion is a consequence of disturbed maternal blood flow prior to impaired spiral artery remodelling [19]. Lastly, dysregulation of a number of key angiogenic factors, such as vascular endothelial growth factor (VEGF) and its receptor, VEGF receptor-1 (Flt-1), have garnered attention as being important contributors of abnormal placental vasculature and the downstream effects [20]. It is for many of the aforementioned reasons that Steegers *et al*. [3] described PE as a “disease of failed interaction between two genetically different organisms”.

Following abnormal placentation, the combined ischemia-induced oxidative stress leads to the release of inflammatory mediators into maternal circulation from the placental intervillous space, including the anti-angiogenic factors endoglin and sFlt-1 [21]. Dysfunction of the all-encompassing endothelium layer which lines the circulatory system is a consequence of the inflammatory response induced by these mediators. For example, soluble Flt-1 (sFlt-1) can absorb free VEGF and placental growth factor (PlGF), thereby effectively blocking downstream vascular development [22]. This can lead to impaired sodium homeostasis of the renal system and other systemic abnormalities [15]. Given the many pathways implicated in leading to PE, a number of studies have been conducted to examine differences in gene expression in PE placentas. These studies have largely been successful in finding significant differential expression of genes in PE individuals within pathways relevant to proposed theories [23]. However, the overarching regulatory mechanisms governing these expression changes remain unclear, and represent a promising area of research towards finding a predictive model to connect the multiple, intertwining routes towards PE.

MicroRNAs (miRs) are small, non-coding RNAs of around 22 nucleotides in length with fundamental roles in regulating diverse biological processes including cell differentiation, apoptosis and development. Their primary regulatory role derives from post-transcriptional gene regulation, where partial or complete binding of miR “seed” regions to the 3’ untranslated region of mRNAs promotes degradation of mRNA transcripts and translational inhibition [24].

Biogenesis of miRs begins with processing pre-miR genes into primary transcripts known as pri-miRs and concludes, after interaction with cytoplasmic components such as DICER, with the mature miR as part of an RNA-induced silencing complex along with one of many catalytic argonaute proteins in the cytoplasm [25]. The mature “guide” strand acts through anti-sense complementarity to target mRNAs, with each miR having the potential to modulate expression of multiple genes, often in a manner specific to tissue type and developmental stage [24].

The relevance of miRs to pregnancy has been explored in studies noting their abundance in human placenta, where precise temporal regulation of gene expression plays an integral part in developmental mechanisms. Further evidence comes from studies revealing the presence of miR biogenesis components, such as Drosha and Dicer, within trophoblast cells [26]. Biogenesis of miRs is essential, as mice models lacking Dicer have deformed vessel formation with embryos dying shortly into gestation [27]. Recently, syncytiotrophoblasts were shown to be a source of miR-containing microvesicles known as exosomes which are released into circulation and protected from RNAse degradation [28]. These placenta-derived, exosomal miRs may be involved in maternal-foetal cell communication, with functions in adapting maternal immune tolerance to the foetus. Indeed, studies have shown exosomes to have roles in immune-related mechanisms such as T-cell signalling [29]. Thus, it appears that miRs play a unique part in placental development and in the progression of pregnancy in general.

Consolidating miRs within proposed mechanisms and clinical phenotypes of PE has been the focus of a cavalcade of studies for almost a decade, since the first major study conducted by Pineles *et al*. [30]. These studies invariably involve use of microarrays, qRT-PCR and next generation sequencing (NGS) techniques to divulge the aberrant expression of miRs in case-control studies consisting of individuals with PE and normotensive individuals with normally progressing pregnancies. While studies initially focused on placental samples from term deliveries, pre-term circulatory samples from PE cases were soon revealed to also harbour detectable quantities of differentially expressed miRs.

However, despite the wealth of studies which have suggested an association of certain miRs with PE, no consensus yet exists on which appear to most significantly contribute towards pathogenesis of the condition, and how they may function in doing so.

Our hypothesis was that miRs have differing expression profiles in PE individuals, and that aberrant expression has a contribution towards pathogenesis of the condition. This study aimed to carry out a systematic literature review detailing the current knowledge encompassing the contributions of miRs to PE pathogenesis, by collecting and comparing results of putatively associated miRs from case-control studies dating back to 2007. Through examining validated targets and pathways of these miRs, a deeper understanding of their involvement within proposed pathogenic domains of interest, and usefulness as prognostic and therapeutic markers can be gained. Additionally, any areas of uncertainty which emerge from a review of current studies will be discussed. In doing so, it is hoped that clarification can be brought to the ambiguous, often contradictory, landscape in this research area and highlight those miRs which show the greatest potential for involvement in PE pathogenesis.

## Methods

### Database search

**Fig 1:**
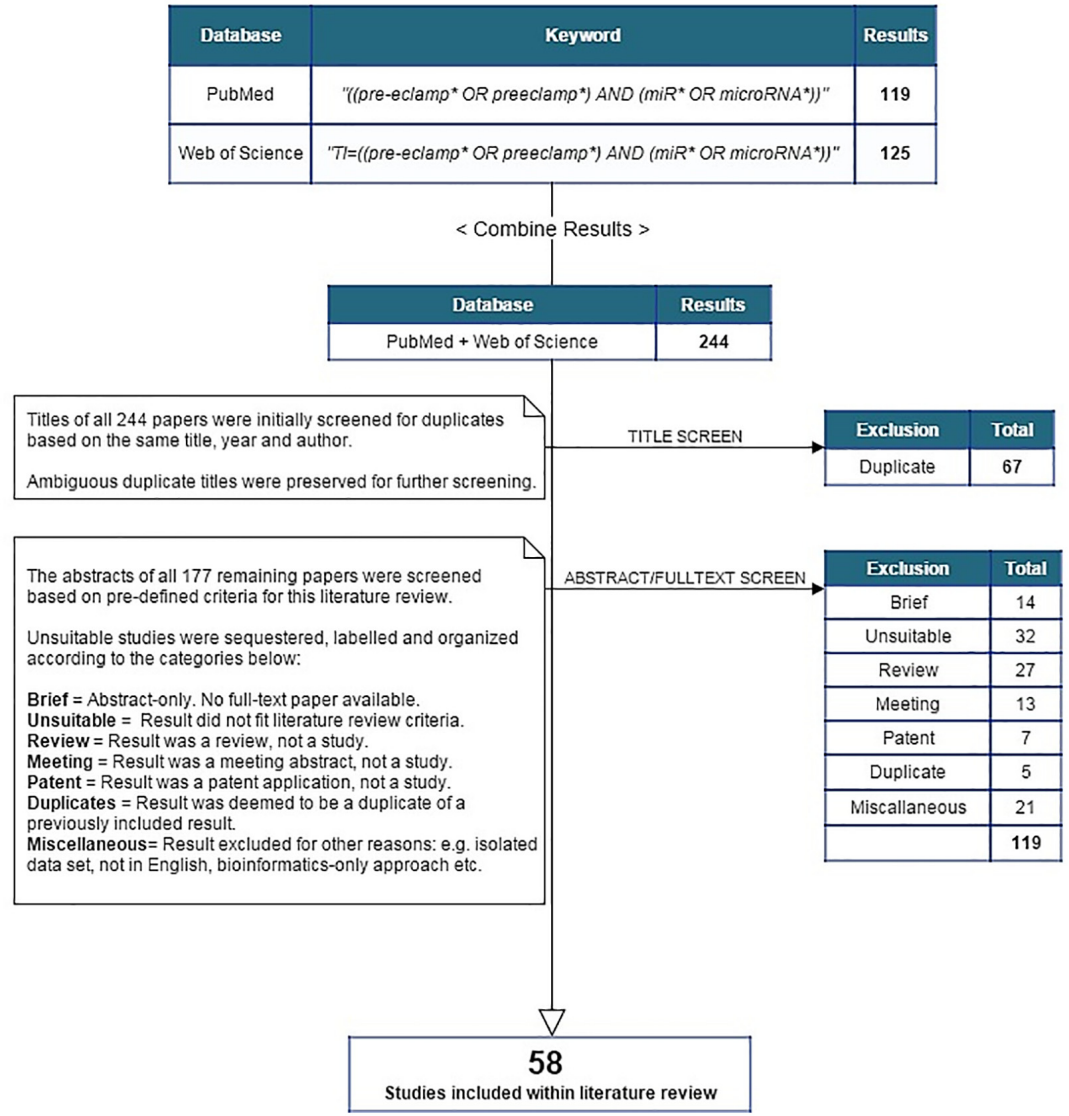
Flow chart denoting the search procedure for this systematic review. This process was repeated multiple times between September 2015 and December 2015.

This systematic literature review queried both PubMed (MEDLINE) and Web of Science databases for studies from the period between March 2007 and December 2015 using the following combinations of keywords: "pre-eclampsia", "preeclampsia", "microRNA" and "miR". The wildcard operator (*) was assigned to each keyword to catch all related terms. Search queries differed according to the database. For Web of Science, only study titles were searched to narrow the result set, which was otherwise too broad in scope. In testing this approach, results returned by use of these single queries included all relevant studies obtained through use of several multi-keyword searches (e.g. “differential”, “expression”) cross-referenced with the primary term of “pre-eclampsia” or “preeclampsia” (Fig 1).

### Study selection

The overall search strategy was predicated upon progressively filtering studies to resolve a set of the most relevant results based upon a pre-defined criterion:

1. The study involves a biochemical investigation of differentially expressed miRs in any sample type (tissue, circulatory fluid or otherwise) between individuals with diagnosed pre-eclampsia and suitable unaffected, normotensive controls using any viable technique (microarrays, qRT-PCR etc.).
2. The study contains details of the methodology used for conducting the investigation. The study includes data on some, if not all, of the discovered differentially expressed miRs. The study has provided this data either within the article itself, or separately in S1 File.
3. The study has been published in a scientific journal and is available in English.

**Fig 2:**
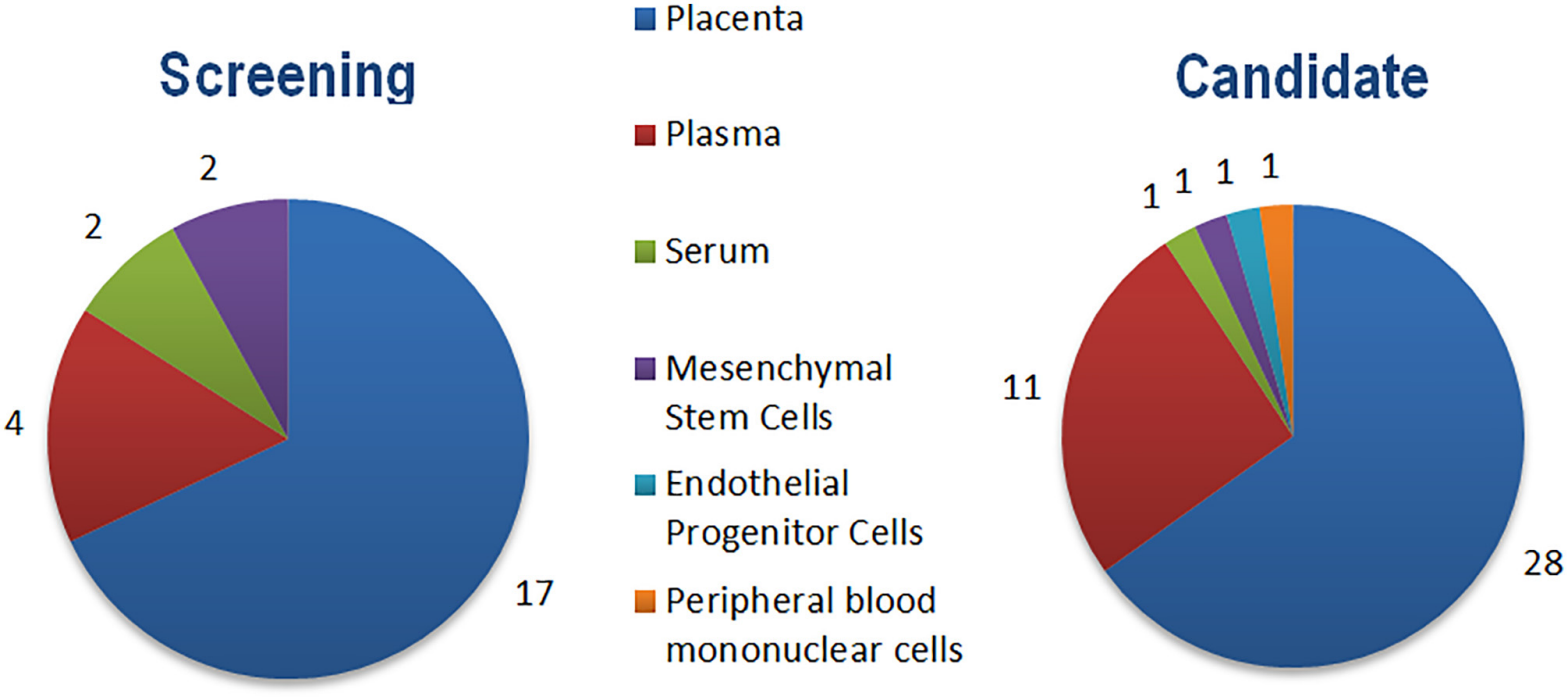
Charts depicting the included screening and candidate studies split by sample type. Some individual studies examined miR expression in multiple sample types.

Studies were initially filtered by screening titles for evidence of duplication. Following this, abstracts were fully screened to ascertain proximity to the above criteria. If an abstract was ambiguous with regards to matching the criteria, or overly vague in content, the full text of the study was subsequently screened to preclude the possibility of removing any potentially pertinent results. Included studies were categorized according to their investigative approach: screening or candidate (Fig 2).

Screening studies involved an ostensibly non-biased approach to profiling miR expression in cases and controls. Candidate studies, by contrast, involved a putative miR or set of miRs for profiling. If a different cohort was used in a screening study for validating a subset of profiled miRs, this validation set was considered to be a separate candidate study. In this way, the combined total of screening and candidate studies surpassed the 58 studies included.

### Data collection

For each study, data was independently extracted regarding miRs determined to be significantly differentially expressed as defined by the study’s criteria for significance, with *p*-value and fold change (FC) serving as the most commonly used parameters. Other comparative elements extracted from each study included cohort characteristics (e.g. sample size, ethnicity), processing method (e.g. RNA extraction kit) and additional information for use in comparing methodologies.

In studies which investigated cases of PE comorbid with other pregnancy-related conditions, such as IUGR, only miRs reported to be significantly differentially expressed between PE and control groups were included—subject to this information being available.

### Data comparison

**Fig 3:**
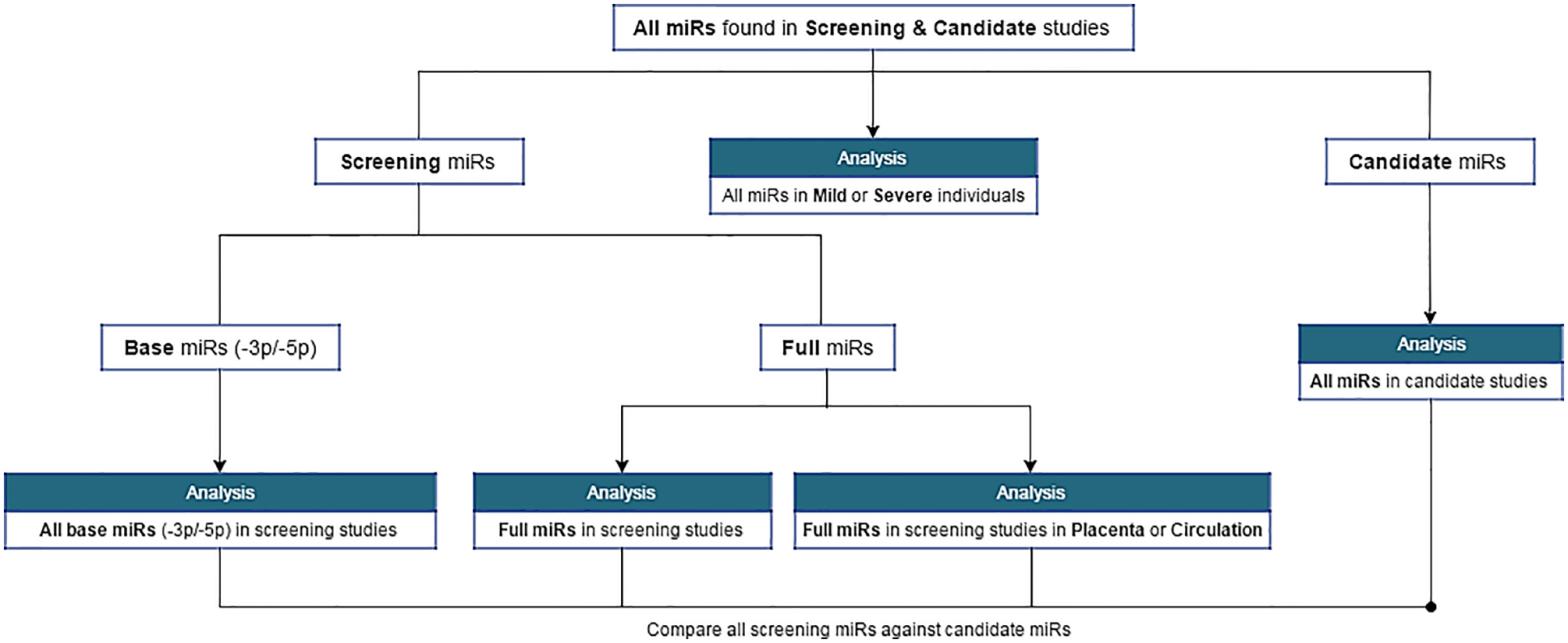
Flow chart depicting the systematic comparison of miRs across studies. Input data was collected from all studies included within the systematic review.

Using the information extracted from each study, an algorithm was written in the PHP scripting language to compare and count miRs from every included study (Fig 3).

While other comparative elements formed the core of discussing methodological differences among studies, data from this algorithm was integral to informing the question of how miRs might contribute to PE pathogenesis. Full data resulting from this algorithm is available separately (S1 File).

For investigating miRs in mPE and sPE cases, the results included were those stated to be significantly differentially expressed exclusively between mPE/sPE cases and normotensive controls, and not between mPE and sPE in case-case comparisons. Furthermore, if the same miR in a study was significantly differentially expressed in both mPE and sPE cases as compared to controls, the miR was only included if its direction of change contrasted between mPE and sPE. This was in order to facilitate the examination of miRs which may discriminate between mild and severe forms of PE.

Studies which stratified PE cases by mild and severe used varying diagnostic criteria—such as ISSHP, ACOG and William’s Obstetrics 23rd edition [9,12,31]. While these overlapped with respect to threshold clinical parameters for systolic and diastolic blood pressure, the requirement and quantity of proteinuria differed between criteria. For example, ACOG stipulates no proteinuria requirement; instead sPE diagnosis is based on the additional presence of systemic involvement [12].

### Data investigation

Several bioinformatics tools were used to further investigate data. Both mirBASE and miRNAVISA were used to identify and explore miR families [32,33]. For investigating miR gene targets and common pathways, DIANA mirPath v3.0 and Targetscan were utilized [34,35]. Ultimately, reported biochemical verification of putative targets through the included and wider literature served as the most reliable and concrete means of ascertaining any significance to PE aetiology.

## Results

### MicroRNAs of interest to PE

**Fig 4:**
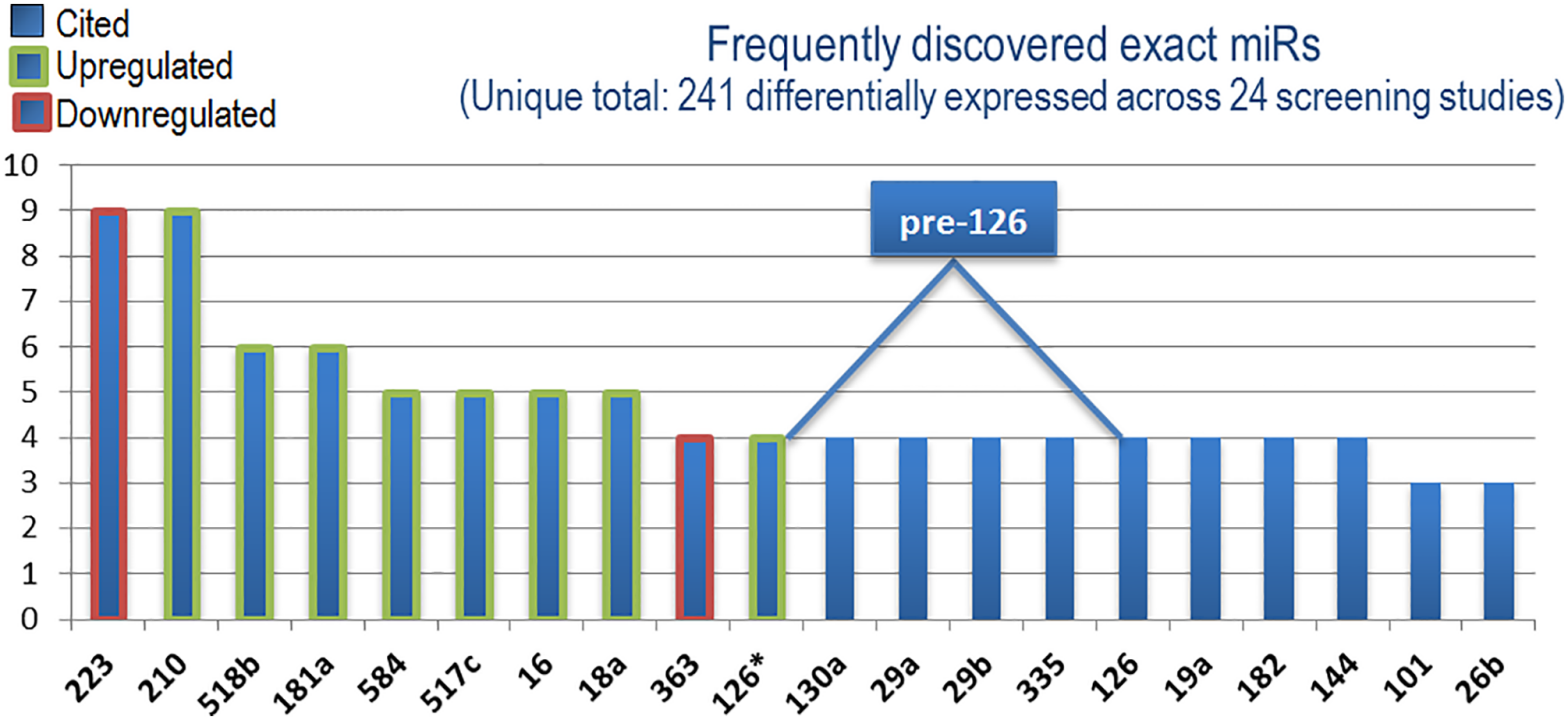
Direction of expression of the most frequently discovered differentially expressed miRs. Border colour represents consensus direction of change across all screening studies for the top 10 miRs.

To address the question of how miRs may contribute to PE pathogenesis, this systematic review first broadly determined those miRs most frequently detected with demonstrable patterns of significant differential expression across all included screening studies (Fig 4 and Table 1).

**Table 1:** List of the 20 miRs most frequently found to be differentially expressed across screening studies of PE.

miR	Validated?^a^	Cited	Validated Targets^b^	References
223	Yes	9	*GZMB*, *STAT3*, *E2F1*, *FOXO1*	[36–44]
210	Yes	9	*HSD17B1*, *ISCU*, *EFNA3*, *HOXA9*	[30,41,44–50]
518b	Yes	6	*FOXN1*, *RAP1B*	[38–41,44,48]
181a	Yes	6	(5p) *BIRC6*, (3p) *CBX5*	[41,44,47,50–52]
584	Yes	5	*PURB*, *CUL2*, (5p) *KPNB1*	[41,44,45,47,50]
517c	Yes	5	*Pyk2*, (3p) *NFIB*	[38,39,42,46,53]
16	Yes	5	*BLC-2*, *CCNE1*	[43,50–52,54]
18a	Yes	5	*TAOK2*, (5p) *INADL*, (3p) *SRF*	[39,41,44,46,53]
363	Yes	4	(3p) *CDKN1A*	[36,41,44,50]
126*		4	*MYC*, (5p) *SLC45A3*	[38,44,46,53]
130a		4	-	[38,39,52,53]
29a	Yes	4	-	[38,39,42,53]
29b	Yes	4	-	[39,43,51,54]
335	Yes	4	-	[47,48,50,51]
126	Yes	4	-	[47,48,51,53]
19a		4	-	[39,41,44,46]
182		4	-	[30,39,47,50]
144	Yes	4	-	[39,44,48,52]
101	Yes	3	-	[36,39,44]
26b	Yes	3	-	[37,39,51]

^a^Validation column states whether or not this miR has been validated by separate qRT-PCR investigation.

^b^Validated targets were collected from candidate studies and use of DIANA-TarBase v7.0 and TargetScan.

*Denotes the minor strand of this miR, as described by the study.

An extensive list of all differentially expressed miRs across studies can be found in S1 File.

While this data provides insight into miRs with suspected PE-related associations, it lends no credence to any causal role in pathogenesis. To examine the potential contributions to PE pathogenesis, two of the most frequently detected differentially expressed miRs, **miR-210** and **miR-223**, will be explored in greater detail through a review of the included candidate studies, and wider literature. Combining *-3p* and *-5p* strand variants of all PE-associated miRs allows for an observation of miR genes commonly associated with PE. Interestingly, both **miR-126** and miR-126* (miR-126-5p) are cited equally as frequently across screening studies and derive from the same precursor gene. As miR-126 has been the subject of early therapeutic investigations, its potential involvement in PE will also be elucidated. Together, these miRs regulate some of the main pathways proposed to account for PE aetiology: ischemia/hypoxia, immunity and angiogenesis/vasculogenesis.

### PE & Hypoxia—miR-210

A paucity of oxygen in the 1^st^ trimester results in the creation of a hypoxic environment which encourages trophoblast proliferation, and leads to increased expression of hypoxia-inducible factor 1α (HIF-1α) in cytotrophoblasts [5]. While hypoxia cedes in normal pregnancy towards the 2^nd^ trimester, persistent ischemia in PE is met with continually high expression of HIF-1α and other hypoxia-inducible elements, such as sFlt-1, in the placenta [55]. Prolonged hypoxia in PE has been attributed to defects in maternal blood flow and/or trophoblast interactions, key factors otherwise responsible for increased oxidative stress and trophoblast invasiveness [3].

Of the 23 hypoxia-regulated miRs detected by Kulshreshtha *et al*. [56], around 18/23 are present in PE across all screening studies included in this review, suggesting the integral nature of hypoxia to pathogenesis (S1 File). Among these, miR-210 has emerged as one of the most prevalent PE-associated miRs. It is increased as much as 13-fold in several hypoxia-induced trophoblast cell lines, with levels maintainable for around 72 hours thereafter [57]. Significant differential expression of miR-210 has been detected in both placenta and circulation in early and late term PE individuals, through screening studies based on microarrays, NGS and qRT-PCR. It is overwhelmingly found to be upregulated, and primarily in individuals with sPE [30,44]. In a case-case analysis, Murphy *et al*. [58] discovered miR-210 levels in plasma to be increased between mPE and sPE samples. Given that more severe forms of PE may be associated with increased hypoxia, and the hypoxic dose-dependent nature of miR-210 expression observed by Fasanaro *et al*. [59], increased hypoxia-driven trophoblast cell debris likely results in higher levels of miR-210 in circulation. Contrary to most studies, both Zhu *et al*. [44] and Enquobahrie *et al*. [60] observed significant downregulation of miR-210 in mPE. This is perhaps due to use of random placental samples and amnion samples in these studies respectively, where different placental regions may differ in levels of hypoxia, made all the less consistent in milder forms of PE.

Hypoxia may contribute to PE pathogenesis through the mediating effects induced by miR-210. The hypoxia-sensitive HIF-1α and NF-κB have been found through ChIP and Luciferase investigations to both bind to the miR-210 promoter and induce expression in a synergistic, co-regulatory manner [57,61]. In trophoblast cells, ectopic expression of miR-210 greatly compromises invasiveness, with the phenotype partially rescuable using high-affinity, anti-miR-210 oligonucleotides [57,62,63]. The mechanisms behind impaired invasiveness appear manifold. It has been suggested that miR-210 inhibits trophoblast invasion via the MAPK and ERK signalling pathways, in a manner inducible by both hypoxia and inflammation [62]. Luo *et al*. [63] demonstrated that overexpression of *KCMF1*, a potassium channel modulator factor and direct target of miR-210 found to be lower in PE individuals, rescued inhibition of trophoblast invasion.

Though a particularly well-studied example, miR-210 represents just one of many PE-related miRs demonstrated to hamper trophoblast invasiveness upon overexpression, including: miR-376c, -155, -34a and -20a [63–66]. Thus, it appears trophoblast invasion is tightly regulated through several, likely overlapping pathways. Whether aberrant expression of miR-210 is an initial cause of defective spiral artery remodelling, or a downstream consequence of the pathogenic cascade, such as an abnormal immunological response, is unclear. Support for an immunological link comes from Kopriva *et al*. [67], who described greatly increased levels of HIF-1α, NF- κB and miR-210 after poly I:C stimulation of Toll-like receptor (TLR) 3 in pregnant mice which developed PE-like symptoms, and in cultured human cytotrophoblasts. TLRs are prevalent in utero-placental tissues where TLR3 recognizes double-stranded RNA viruses of the kind which poly:IC simulates. While TLR3-activated pregnant mice developed hypertension, TLR3 knockout mice induced with poly:IC did not, nor did levels of miR-210, HIF-1α and NF- κB increase significantly [67]. In combination with the suggested increased risk of PE in women with viral infections, and the greatly increased miR-210 expression in activated T-cells, this lends credence to a miR-210-mediated immunological pathway involved in PE pathogenesis [68,69].

Hundreds of gene targets have been predicted for miR-210 through *in silico* searches, but as with miRs in general, the precise function is largely dependent on the cellular context.

Lee *et al*. [70] verified iron-sulfur scaffold homologue (*ISCU*) to be a target of miR-210, finding decreased levels in both PE and SGA in line with increased miR-210 expression under hypoxic conditions and consistent with studies of ISCU in relation to cancer and cardiovascular biology. ISCU is one of many proteins which make up the iron-sulfur clusters involved in the electron transport chain and oxidation-reduction reactions within mitochondria. In this way, miR-210 may be an important contributor towards the long-recognized increased oxidative stress and mitochondrial abnormalities in PE, the latter of which has been shown to be ameliorated by using miR-210 inhibitors [71,72].

Kopriva *et al*. [67] demonstrated signal transducer and activator of transcription 6 (*STAT6*) to be a direct target of miR-210, observing decreased IL-4 expression coinciding with decreased STAT6. Ishibashi *et al*. [46] found that miR-210 targeted the enzyme 17-beta-hydroxysteroid dehydrogenase (*HSD17B1*) responsible for catalysing the production of estrone into 17β-estradiol. The level of 17β-estradiol is known to be lower in women with sPE, and is expressed exclusively in the placenta [73]. This is one of many estrogen-catabolizing enzymes with noted dysfunction in PE individuals, the prototypical example being catechol O-methyltransferase [74]. Lastly, both Fasanaro *et al*. [59] and Zhang *et al*. [57] verified Ephrin-A3 (*EFNA3)* as a target of miR-210. Among functions in embryonic development, Ephrins are important for vascular development and cell migration, particularly in the cardiovascular system. Inhibition of miR-210 and use of an *EFNA3* allele lacking the miR-210 binding site both successfully abrogated the downregulation of Ephrin-A3 under hypoxic conditions. With these targets in mind, and the elucidated feed-forward loop which is suggested to exist with HIF1α, miR-210 appears to be an important player contributing to PE pathogenesis via hypoxia-induced factors [75].

### PE & Immunology—miR-223

Given the connections between PE and immunological adaptation, it is interesting that one of the most prevalent miRs across screening studies is often described in relation to immune tolerance and the inflammatory response, miR-223. In as much as miR-223 is studied in other conditions, comparatively little is known about its contribution to PE. It is generally found to be downregulated in sPE and eoPE, both in placental and circulatory samples (S1 File).

The highly-conserved gene encoding miR-223 is located on the X-chromosome, where the promoter has demonstrated specificity for the haematopoietic system with roles which include regulation of haematopoetic cell differentiation, quelling inflammatory response during infections and platelet adhesion [76,77]. While myeloid cells, particularly granulocytes, show high levels of miR-223, expression has also been noted in murine macrophages and resting natural killer cells—where levels in both decrease upon cytokine activation [78–80]. Natural killer cells are abundant in the placenta during early pregnancy and have suggested roles in establishing tolerance to the newly implanted blastocyst [17]. Given this, decreased placental miR-223 in early pregnancy may serve as an early predictive marker of aberrant natural killer cell-related tolerance. In placenta, miR-223 was found to be expressed in both chorionic and basal plates, in somewhat stark contrast to many other PE-associated miRs such as miR-210, which appear to be preferentially expressed in either region [41]. The seemingly ubiquitous nature of miR-223 may partially explain its prevalence in studies using random placental sampling.

Downregulation of miR-223 has been noted in obesity, inflammatory diseases, cancers and autoimmune diseases such as type 2 diabetes [76]. In order to investigate possible pathways and target genes for miR-223 of relevance to PE, the DIANA mirPath v3.0 tool was employed. Validated targets were found in pathways predictably relating to cancer, but also FoxO signalling—including *STAT3* in both pathways and forkhead box protein (FOX) O1 in the latter.

FOXO1 localizes to the trophoblast layer of the choriodecidua and is a transcription factor involved in regulating key cellular processes such as oxidative stress and apoptosis [81]. While direct targeting of the 3’UTR of *FOXO1* mRNA by miR-223 has been demonstrated in cancer cell lines, a significant decrease in FOXO1 expression has only been observed in mPE, and not sPE [82,83]. STAT3 is a part of the STAT pathway which can be activated by both pro- and anti-inflammatory cytokines, such as IL-6 which is itself increased after STAT3 phosphorylation [80]. Phosphorylated STAT3 under hypoxic conditions has been shown in multiple cancer cell lines to stabilize and simultaneously bind with HIF-1α to separate sites on the VEGF promoter, in order to maximally induce expression [84,85]. Interestingly, the PE-associated miR-20b, part of the miR-106a/363 cluster, has also been shown to target *STAT3* and *HIF1A*, and no studies have found differential expression of both miR-20b and miR-223 together in PE [86,87]. Similarly, miR-20a is upregulated in PE and directly targets *FOXA1*, perhaps suggesting similar pathogenic pathways among these PE-associated miRs [65]. Chen *et al*. [80] found that overexpressing miR-223 downregulated both IL-6 mRNA and protein production in poly I:C-stimulated, TLR-activated macrophages by directly targeting murine *STAT3* for translational inhibition.

Further exploration of the proposed IL-6/miR-223/STAT3 pathway using IL-6 specific antibodies found the hugely reduced miR-223 levels after TLR-activation to be likely mediated by IL-6, which in so doing, further increased IL-6 production in a positive regulatory manner. Increased IL-6 has been shown to enhance vascular dysfunction and promote macrophage infiltration, with downstream effects on cytotrophoblast invasiveness [88]. Additionally, treatment of placental explants with IL-6 increased trophoblast shedding, with cells showing reduced caspase activity [89]. Given immunohistochemical evidence of increased IL-6 levels in PE individuals within decidual and cytotrophoblast cells, as well as in circulation, this may represent a novel pathway for miR-223 in PE pathogenesis [88,89].

A recent study by Schjenken *et al*. [90] suggested that expression of miR-223 may be induced in tissues of the female reproductive tract after exposure to seminal fluid. Absence of miR-223 then, could compromise immunological adaptation around conception. In the study, *miR-223* null mice had substantial changes in immune and inflammatory gene expression, lower T-regulatory cell counts and as pregnancy progressed, a lower fetal:placental weight ratio. Interestingly, this perhaps lends credence to a miR-223 role in prior observations of maternal tolerance to paternal alloantigens being dependent on exposure to seminal fluid, as well as the suggested increase in PE risk in nulliparous women below a certain age threshold [91]. Lastly, there is compelling evidence that miR-223 may be involved in maintaining the quiescence of endothelial cells, where lower expression is met with the promotion of angiogenic status via increased β1-integrin expression [92]. Hypoxia has been shown to downregulate a key upstream regulator of the miR-223 promoter, C/EBP-α, through HIF-1α [76,93]. Thus, lower expression of miR-223 in PE could be a compensatory mechanism to reduce endothelial quiescence and subsequently increase angiogenesis. With the wealth of potential connections to PE pathogenesis in mind, further exploration of miR-223 in relation to PE is warranted.

### PE & Angiogenesis—miR-126/126*

Impaired angiogenesis is a pillar of PE pathogenesis, and many PE-associated miRs are known to have putative targets related to angiogenesis [51]. Of these, miR-126 represents one of the most intimately connected, angiogenesis-related miRs. Encoded by the 7^th^ intron of the *EGFL7* gene in mammals, miR-126/126* is highly-conserved, being characterized in both mice and zebrafish [94]. Endothelial cells, including those of the human umbilical vein, are highly enriched for miR-126/126* and pivotal roles for this miR have been described in modulating aspects of endothelial homeostasis—including enhancing the pro-angiogenic functions of VEGF and fibroblast growth factors via the MAP kinase pathway [95]. That 40% of *miR-126* knockout mice have an embryonic lethal phenotype while surviving mice show compromised wound healing reflects the potent, albeit non-essential and context-dependent nature of miR-126 expression in early development, and in maintaining angiogenic homeostasis thereafter [95].

Significant differential expression of both miR-126 and miR-126* has been detected across screening studies, but with no clear consensus on directions of change in PE (Table 2).

**Table 2:** List of studies detecting differential expression of miR-126* or miR-126, with their respective directions of change.

Study type	Study	miR	Sample type	Direction of change (PE sub-type)
Screening	[38]	126*	Placenta	Increase (higher in mPE)
Screening	[51]	126	Placenta	Increase (sPE)
Screening	[46]	126*	Placenta	Increase (mostly loPE)
Screening	[47]	126	Placenta	Decrease (sPE)
Screening	[48]	126	Serum	Decrease (sPE)
Screening	[53]	126,126*	Placenta & Plasma	Increase^a^
Screening	[44]	126*	Placenta	Decrease (sPE)
Candidate	[96]	126	Placenta	Decrease
Candidate	[97]	126	Placenta & EPCs^b^	Decrease

^a^In this study, miR-126 and -126* were upregulated in both placental and plasma samples with varying levels of expression between sample types and fold change differences between mPE and sPE.

^b^EPC = Endothelial progenitor cell.

A general consensus towards increased miR-126* expression appears evident in placental samples. For example, NGS studies by Guo *et al*. [38] and Yang *et al*. [53] both detected increased miR-126* in mPE, while increased miR-126* in the study by Ishibashi *et al*. [46] was detected in a cohort mainly consisting of individuals with the assumedly milder, loPE. Additionally, in determining expression from both mPE and sPE individuals, Zhu *et al*. [44] detected decreased miR-126* in only the latter. Although functional differences between both mature strands are not well understood, miR-126* may play a part in the arterial injury response by targeting *Dlk1*, an inhibitor of Notch1 which negatives regulates endothelial cell proliferation [98]. Mechanisms governing selection of the functionally active strand are unclear, however. That expression of both strands in a non-mutually exclusive manner has been observed in PE is intriguing, given the expected inhibitory outcome. An explanation for this may arise from evidence of different argonaute complexes integrating either miR strand, allowing for coinciding functional roles [99]. Differential ratios of miR-126 strand expression in PE classifications may reflect differences in pathogenetic mechanisms between mPE and sPE, or in the adaptive response to varying degrees of angiogenic insult—perhaps through miR arm switching [100].

Although unclear from screening studies alone, a consensus on decreased miR-126 expression appears evident from candidate studies of this miR (Table 2). As blood flow can activate a pathway mediated by the zinc-finger transcription factor KLF2A towards inducing miR-126 expression in endothelial cells, the ischaemic consequences of PE could lead to an overall decrease in miR-126 expression which correlates roughly with severity of the condition [101]. Evidence of the capacity for hypoxia to reduce miR-126 expression exists for RF/6A retinal cells, and disturbed flow has been noted to also reduce levels of miR-126* [98,102]. An imbalance of angiogenic factors in PE, including a reduction in pro-angiogenic VEGF-A, which has roles in endothelial cell survival, may work in tandem with possible increased hypoxia in more severe PE to reduce the density of endothelial cells and subsequently, miR-126 expression [103]. Perhaps supporting this, Yan *et al*. [97] noted a decreased number of endothelial progenitor cells (EPCs) in PE patients, which could be moderately correlated with miR-126 expression.

How miR-126 acts to influence the dynamics of angiogenesis has been reasonably well-studied outside of PE. For example, in chronic kidney disease, both miR-126 and miR-223 have been noted to increase in the aortas of mouse models throughout disease progression [104]. In the context of PE, a candidate study using qRT-PCR by Hong *et al*. [96] found significantly lower miR-126 levels in placental samples from 115 PE cases, which positively correlated with lower *VEGF* mRNA levels. Validated 3’ UTR targets of miR-126 include phosphoinositide-3-kinase regulatory subunit 2 (*PIK3R2*) and sprouty-related EVH1 domain—containing protein 1 (*SPRED1*), both integral components of the VEGF signalling pathway [105]. PIK3R2 is known to be important in negatively regulating phosphatidylinositide 3-kinase (PI3K) and its downstream target Akt, which it phosphorylates in a VEGF-induced manner with effects on angiogenesis [105]. In a candidate study of miR-126 in EPCs, Yan *et al*. [97] found decreased PIK3R2 expression after transfer of miR-126 mimics, with increased levels of mRNA and protein for both PI3K and Akt. The connection was reinforced by a *PIK3R2* loss-of-function study which revealed similar effects to the mimic.

Further inoculation of rat placentas with antagomir-126 revealed a reduction in the size and weight of placentas, as well in microvessel density [97]. Rodents have hemochorial placentas, much like humans, and share the placental processes of trophoblast invasion and artery remodelling. In a later animal model study, miR-126 was decreased by 57% in the placentas of rats administered with the nitric oxide synthase inhibitor L-NAME, which effectively reproduces the hypertensive and proteinuric effects of PE [106]. Interestingly, overexpressing agomir-126 alleviated many symptoms of placental dysfunction in PE rats, leading to better pregnancy outcomes (e.g. higher microvessel density and pup/placenta weight) while having no significant effect on PIK3R2 protein expression or on blood pressure. Regulation of blood pressure and PIK3R2 expression by other factors likely accounts for some of these apparent discrepancies. This may include additional targets of miR-126 in PE which are imperfectly emulated in the L-NAME model, and which could result in compounded downstream effects on blood pressure and PIK3R2 expression.

The authors suggested that variation in blood pressure within the small sample group and lack of optimized treatment protocols may also be implicated. While hinting at the promise and limitations of miR-126 for use in a therapeutic context, it is unknown how evaluations of efficacy in rodent models would translate into the distinctly human manifestation of PE. Lastly, miR-126 has been shown to regulate Ephrin-B2 (*EFNB2*), an inhibitor of MAP kinase signalling with roles further downstream of VEGF [105]. *EFNB2* represents a common target of the angiogenesis-related miR-20b, which shares a seed sequence with miR-17 and -20a and have all similarly been found to be differentially expressed in individuals with sPE [87]. Given the attention miR-126 has already received, subsequent elucidation of other downstream targets and effects will allow for the already promising aspects of miR-126 in therapeutic intervention to be thoroughly explored.

### Familiar Territory—The C19MC Cluster

**Fig 5:**
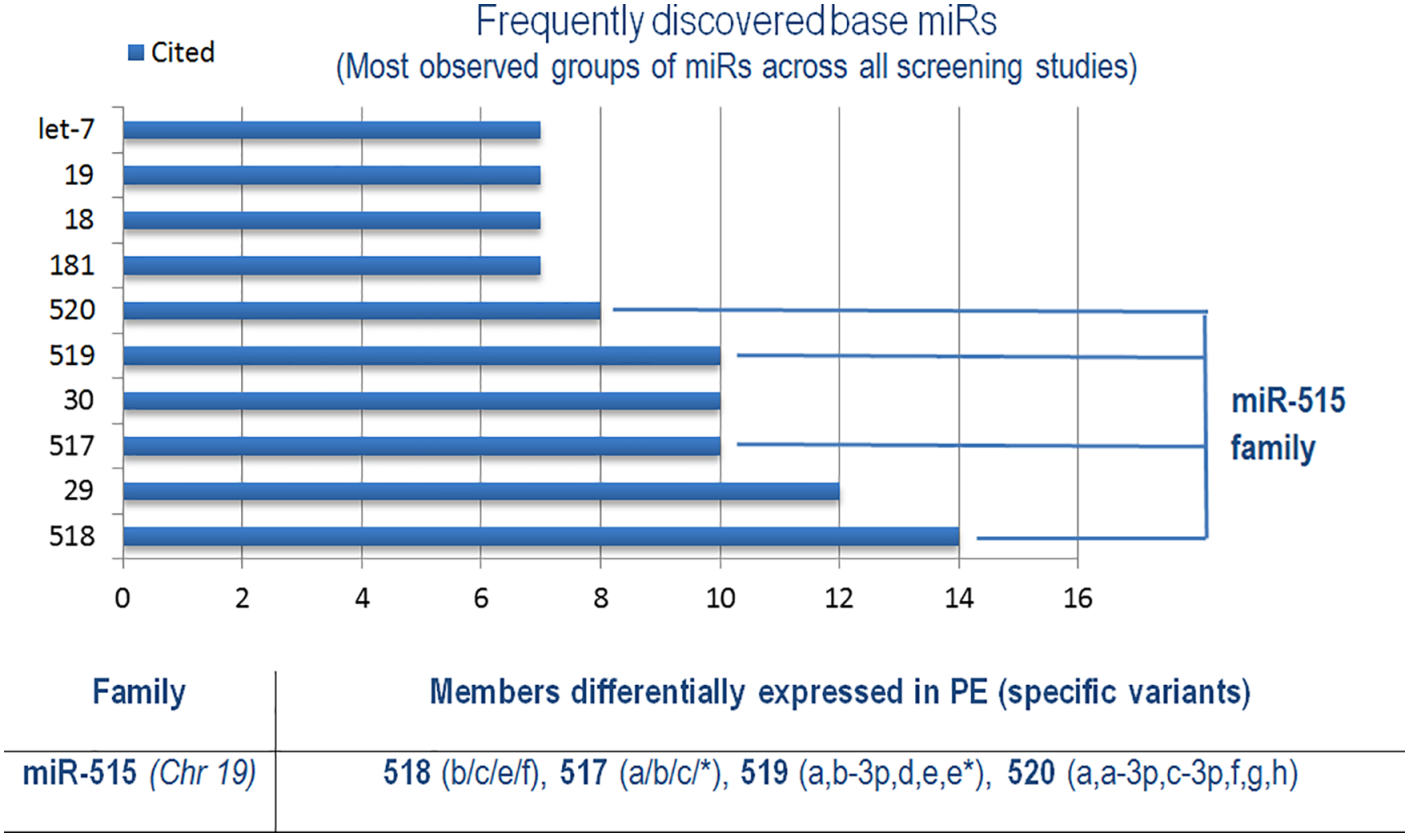
Collection of base miR groups containing putatively related, differentially expressed members across all included screening studies. Group which consisted solely of the same repeated exact miR entity (e.g. miR-181a) were removed. The miRs were grouped together into families using mirBASE and miRNAVISA. See S1 File for a full list of specific miRs belonging to these groups and study references.

Organization of miRs into families based on common seed sequences with the assumption of related targets has proven useful in cancers in informing patterns of interaction between related miR entities [107]. To determine miR families with relevance to PE pathogenesis, the base miR entities with markers of relatedness (e.g. miR-29a, -29b) and strands references (-3p, -5p) collected together were split into groups across screening studies (Fig 5).

In so doing, 11 independent studies discovered members of the miR-515 family belonging to the chromosome 19 miR cluster (C19MC) to be among the most represented across all included screening studies (S1 File). The primate-specific C19MC is a paternally imprinted cluster consisting of around 46 pre-miR genes spread across a 100kb region, with the miR-515 family comprising one of two large sub-groups within [108]. Members of the C19MC cluster are highly enriched in placenta, particularly in term trophoblast cells where they comprise a significant proportion of cellular miR content and demonstrate increased expression from 1st to 3rd trimester [109,110]. Increased expression is paralleled in maternal circulation and C19MC miRs compose the majority of exosomal miRs, where they could be important players in cell-cell communication [109,111]. In regards to this, the extracellular-packaged C19MC miRs in circulation have recently been shown to exhibit anti-viral activity in recipient cells, somewhat reminiscent of prokaryotic RNAi immune mechanisms [112]. Given the prominent expression of C19MC in normal placental physiology, it is expected that aberrant regulation could have significant contributions towards PE pathogenesis.

This cluster has been the focus of several candidate studies, which generally conclude C19MC members to be upregulated in cases of sPE and eoPE, in both plasma and placental samples [113–116]. In normal pregnancy, miR-517b and miR-519a are suggested to be important for regulating trophoblast proliferation in early gestation [117]. Anton *et al*. [114] found that transfection of miR-517a/b, which have identical sequences, and miR-517c to first-trimester extravillous trophoblasts dramatically decreased invasiveness—also observing that these miRs appeared to be increased under hypoxic conditions. While validated targets are conspicuously lacking for miR-517a/b/c, increased expression was associated with increased sFlt-1 levels along with the anti-angiogenic cytokine, TNFSF15. Little is known about the involvement of miR-518b in PE, despite considerable evidence of its aberrant expression (Table 1). In embryonic carcinoma cells treated with BMP-2 to adopt an epithelial phenotype, miR-518b was shown to directly target *FOXN1*—a transcription factor critical for thymus development and epithelial differentiation [118]. Knockout mice for the *Foxn1* gene are phenotypically hairless ("nude") and severely immunodeficient [119]. There is also indication from an oncological study that upregulation of miR-518b is associated with inhibited cell migration in chondrosarcoma cells [120].

Yang *et al*. [53] found miR-519d to be among the highest proportionally differentially expressed miRs in PE cases, and corroborated the high expression of C19MC members in plasma samples. Additional studies have reinforced upregulation of miR-519d in circulatory samples from PE cases [39,42,116]. This miR has been associated with obesity, and a candidate study of miR-519d-3p in placenta by Ding *et al*. [115] suggested that by directly targeting the 3‘UTR of matrix metalloproteinase-2 (*MMP-2*), one of many extracellular matrix proteases, miR-519d-3p was able to suppress trophoblast invasion and migration [121]. Further investigation found MMP-2 to be reduced in the placentas of PE individuals and negatively correlated with miR-519d-3p, though the effect of miR-519d-3p mimics on *MMP-2* luciferase activity after transfection appears modest. These results extend findings by Xie *et al*. [122] who observed that miR-519d could reduce expression of *Cxcl6*, *Foxl2* and *NR4A2* transcripts, where Cxcl6 and NR4A2 have previously described roles in cell migration in neutrophils and mesenchymal stromal cells respectively.

Lastly, after exposure of primary human trophoblasts to hypoxia, Donker *et al*. [109] found miR-520c-3p to be selectively downregulated, with no significant effect on other C19MC miRs. This was reinforced in a screening study by Betoni *et al*. [36], perhaps suggesting a link between hypoxia and downregulation of particular C19MC members. However, comparatively few studies have since corroborated differential expression of miR-520c-3p in PE, with miR-517c and miR-518b being the most widely detected PE-associated C19MC members across screening studies (S1 File).

As the C19MC cluster is expressed from the paternal chromosome and epigenetically-regulated through an upstream CpG-rich site, increased C19MC expression throughout normal pregnancy, and aberrant upregulation in PE, may be due to progressive loss of methylation [123]. Indeed, significant hypomethylation at several gene loci has been associated with eoPE and use of a demethylating agent in carcinoma cells restores C19MC expression [123,124]. Upregulation of C19MC members could then lead to a combinatorial effect on inhibition of invasiveness. With this in mind, differential expression of the entire C19MC has been suggested to account for differences between villous and extravillous trophoblasts, where the latter shows lower expression of the cluster and is responsible for invading the maternal decidua in early gestation [122]. Overall, the C19MC cluster and families contained within represent an incredibly promising and critically under-researched area, both for PE and understanding normal placental development. Additionally, from an evolutionary perspective, deeper study of the C19MC could provide interesting clues towards advancing an understanding of how miR clusters have contributed to the development of the primate lineage.

## Discussion

### Methodology comparison

This literature review accounted for 241 unique miRs across the included screening studies. Of these, approximately 20% of the combined total were present in more than one study and verified by qRT-PCR either within the same screening study, or independently (Table 3).

**Table 3:** List of PE-associated miRs from included screening studies which were found to be differentially expressed in more than one independent study and validated by qRT-PCR.

miR	References
223	[36–44]
210	[30,41,44–50]
518b	[38–41,44,48]
181a	[41,44,47,50–52]
584	[41,44,45,47,50]
517c	[38,39,42,46,53]
16	[43,50–52,54]
18a	[39,41,44,46,53]
363	[36,41,44,50]
29a	[38,39,42,53]
29b	[39,43,51,54]
335	[47,48,50,51]
126	[47,48,51,53]
144	[39,44,48,52]
101	[36,39,44]
26b	[37,39,51]
25	[37,39,48]
1	[44,45,50]
195	[41,44,51]
214	[41,43,51]
519e*	[41,44,46]
152	[44,47,48]
519d	[39,42,53]
221	[39,43,52]
30a	[39,43,54]
24	[39,40,52]
542-3p	[41,42,44]
199b-5p	[36,43]
140-5p	[36,43]
342-3p	[37,52]
296-5p	[37,48]
26a	[37,52]
141	[38,51]
149	[53,125]
20b	[51,87]
193b	[41,46]
526b	[40,46]
20a	[46,50]
377	[44,47]
519a	[39,42]
520h	[39,42]
100	[39,43]
19b	[39,50]
495	[40,43]
30a-3p	[41,44]
296	[41,44]
218	[41,44]
411	[41,44]
136	[42,43]
18b	[44,53]

*Denotes the minor strand of this miR, as described by the study.

The evident lack of congruity between results creates considerable doubt when assigning a role for the huge variety of putative miRs in the pathogenesis of PE with any degree of certainty.

Differences in methodology likely play a significant part in creating variability between results, particularly in areas such as the definition of PE, cohort characteristics, sampling technique, sample processing and data normalization. These suggested categories of methodological incongruity will be explored.

### Defining pre-eclampsia

Classification systems for PE employed by major organizations differ in ways that could meaningfully impact studies. Areas of contention include: inclusion of non-proteinuric gestational hypertension as PE, definition of severe hypertension, and relative consideration given to eoPE [3]. In this review, it was noted that PE definitions were relatively consistent throughout, with most studies opting to use ISSHP diagnostic criteria—even if not directly stated. Furthermore, screening studies typically stratified PE cases by mild/severe and around half of all studies profiled miR expression in only sPE cases, with average GAs at delivery ranging from 33–40 weeks. This highlights the variability in severity within sPE definitions alone. To determine whether a selection of miRs could help delineate PE severity, all studies investigating differential expression in mPE and sPE cases were collected to examine miRs detected exclusively in either so far. Some of the most frequently detected miRs in sPE cases whose aberrant expression had not been determined in mPE cases at the time of this review included: miR-181a, -223, 195, -16, -18a and -363 [37,39,41,43,44,47,50–52,58,87,126,127].

It is worth noting that the absence of any putatively severe-associated miR from mPE does not constitute its exclusive differential expression in sPE. However, it could indicate a correlation between a detectably higher FC of the miR, and more severe forms of PE. For example, miR-181a is one of the most independently validated miRs often associated with sPE, and has been shown to modulate T-cell sensitivity/selectivity as well as enhance IL-6 expression in mesenchymal stem cells (Table 3)[126,128]. Most screening studies observing upregulation of miR-181a, also noted upregulation of miR-210 [41,44,47,50,129]. These studies predominately involved sPE cases, and a trend towards earlier delivery at around 34 weeks can be observed in those reporting average GAs at delivery. Following this, the two studies finding miR-181a to be upregulated without miR-210 involved late-onset sPE, with PE cohorts from both having later average GAs at delivery of around 37 weeks [51,52]. This could indicate upregulation of miR-181a coinciding with miR-210 to be a marker of more severe forms of sPE—perhaps implying a particular pathogenetic route, e.g. immunological. That downregulation of miR-181a has also been determined in placentas from pre-term deliveries at <35 weeks suggests that aberrant expression of miR-181a is implicated in different pathogenic mechanisms relating to severe pregnancy outcomes [40]. However, unlike miR-181a, differential expression of miR-210 has also been observed in mPE—perhaps due to the sensitivity of its expression in a hypoxic environment as previously discussed.

Members of the miR-15 precursor family, such as miR-16 and miR-195, are among the most frequently associated with sPE and have been independently verified in multiple studies (Table 3). Candidate studies revealed *VEGFA* to be a probable target of miR-16, which has previously been shown to regulate progression of the cell cycle and ameliorate decidual mesenchymal stem cell proliferation in sPE, suggesting roles in impaired angiogenesis [43,54]. On the other hand, miR-195 was found to modulate Activin/Nodal signalling through targeting *ActRIIA*, and in so doing, promote invasiveness in a trophoblast cell line [127]. Given that the effect on invasion is relatively minimal, other targets of this miR may also contribute towards pathogenesis.

The inclusion of GA into PE classification is critical. A significant increase in maternal mortality has been associated with eoPE, with the symptomatic chasm between eoPE and loPE such that separate pathogenetic mechanisms with overlapping phenotypes have long been suspected [130,131]. Although only 5–20% of cases are considered eoPE, in stark contrast to more than 80% for loPE, these cases typically represent the most severe end of the symptomatic spectrum [132]. In assigning certain pathological features to eoPE, however, the co-existence of IUGR in most eoPE cases muddies the water considerably, as many of the most severe symptoms in eoPE are also observed in isolated cases of IUGR [132]. Whether or not the included cases of sPE in most studies also represent eoPE is uncertain, but the larger overall average GAs at delivery may indicate severe loPE forms. A lack of consistency in eoPE and loPE definitions was seen across all studies, with eoPE defined as either the onset of PE symptoms at <34 or <32 weeks, or by thresholds values for GA at delivery.

A screening study by Weedon-Fekjær *et al*. [49] detected a general over-representation of aberrantly expressed miRs in eoPE (as defined by delivery at < 34 weeks), focusing upon downregulation of miR-1301 which showed a correlation with foetal weight percentile in eoPE cases (3 of which included IUGR). In a separate candidate study, expression of miR-517a/b/c, members of the C19MC, were all significantly upregulated in eoPE in a manner that appeared to correlate with severity [114]. Lastly, Gao *et al*. [133] found miR-675, encoded in the paternally-imprinted and hypomethylated *H19* gene, to be downregulated significantly in eoPE. This miR was demonstrated to inhibit trophoblast cell growth, possibly by targeting Nodal Modulator 1 (*NOMO1*), an antagonist of the Nodal signalling pathway which is important for regulating cell growth [133].

Where the contribution of a miR cannot be attributed to PE alone, as in studies involving cases of PE co-morbid with conditions such as SGA, animal models can serve as a powerful means to help resolve ambiguity. An example of this is in the screening study by Pineles *et al*. [30], who detected differential expression of miR-155 in a cohort of PE individuals complicated with SGA, but not in the group with PE alone. The PE+SGA group also had notably higher average values of systolic and diastolic blood pressure than the exclusively PE cohort. The clarification of an association between miR-155 and PE arose from an animal study conducted by Liu and Yang [134], who detected aberrant expression of miR-155 in L-NAME rat models of PE.

### Cohort characteristics

Among case and control groups from included studies, matching of characteristics generally occurred for both GA at delivery and maternal age. Nulliparity was the most explicitly stated characteristic to be additionally matched across screening studies. However, as with other characteristics (e.g. ethnicity and BMI) matching infrequently reached significance after use of statistical testing, such as the Mann-Whitney rank-sum test. The most consistent differences in PE cases as compared to controls, outside of diagnostic criteria, were increased nulliparity, decreased GA at delivery and decreased foetal birth weight.

Ethnic composition was rarely indicated in studies. A study by Goodwin and Mercer [135] found African-American women to be more likely to present with severe and persistent hypertension. This may relate to findings of a pre-disposing polymorphism (G) of the human leukocyte antigen occurring at higher frequencies within African-American populations [136]. In the study by Pineles *et al*. [30], PE cases had among the lowest average GAs at delivery for the sample size, and consisted of ethnically Black individuals. By contrast, a recent study found ethnically Chinese individuals to have a lower prevalence of PE, as compared to Caucasians [137]. Given this, and knowledge of population differences in allele frequencies of SNPs which impact miR expression, the population included may influence variability in results [138]. In combination with environmental and socioeconomic variations within populations, this could meaningfully impact comparability between studies.

**Fig 6:**
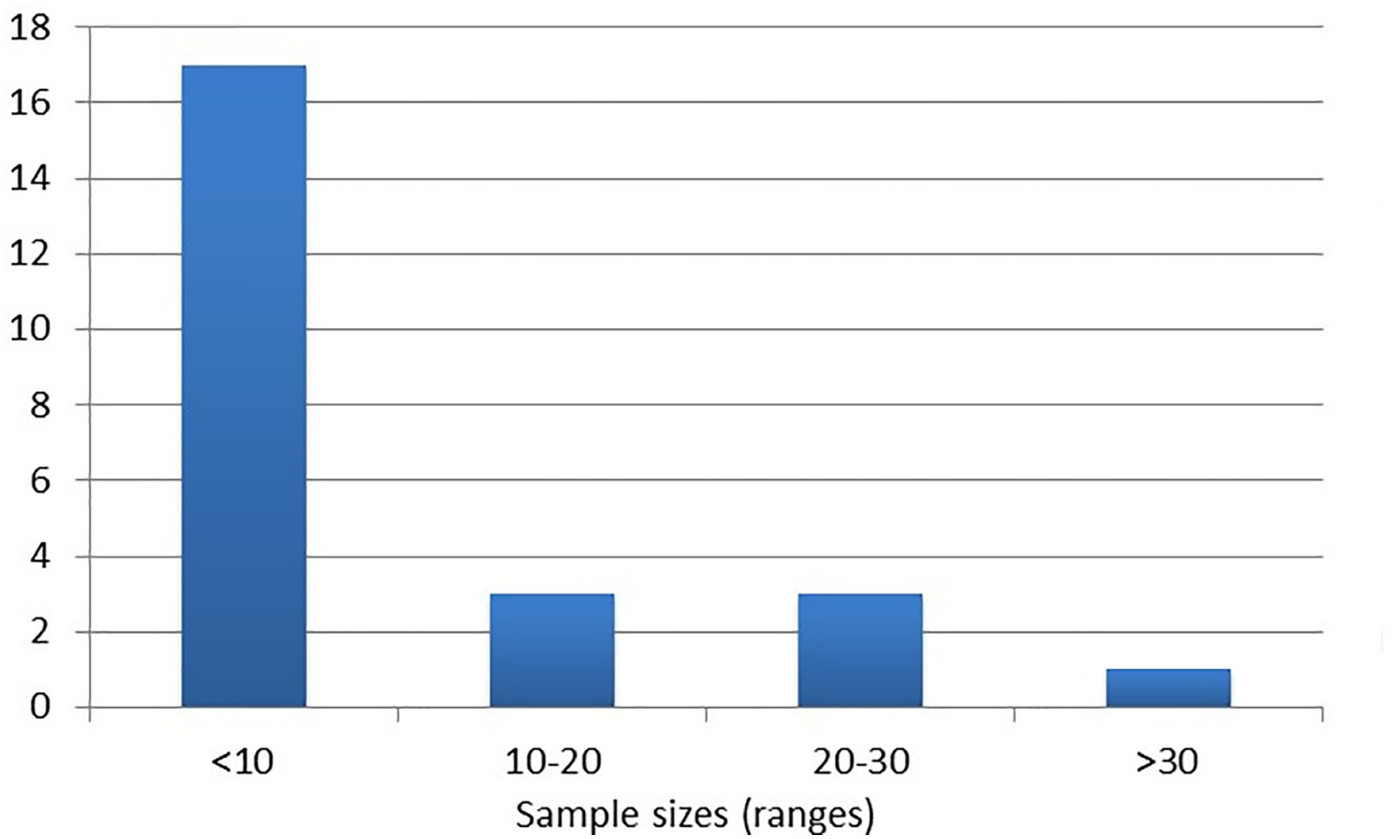
Sample size groups used across all included screening studies.

The vast majority of screening studies used PE cohort sizes of less than 10 individuals, particularly those involving microarrays (Fig 6).

In considering the apparent heterogeneous nature of PE pathogenesis, with multiple risk factors and inherent genetic/environmental variability, these smaller cohort sizes may have a reduced power to detect genuine differential expression of miRs.

Studies would therefore benefit from prior analysis to determine recommended sample sizes for achieving the necessary statistical power. Lastly, few studies mentioned prior or on-going treatment for PE in included cohorts, and it is unclear how treatment options, such as use of anti-hypertensive medications, could impact expression patterns.

### Sample, technique and timing

Technical and ethical reasons necessitate placental samples being taken after delivery, and it is unknown how accurate miRs detected at this stage are in representing earlier pathological mechanisms. Many studies have highlighted the tissue-specific, cell-specific and temporal nature of miR expression during placental development [110,139]. This is particularly evident between the 1st trimester and term periods, where changes in oxidative stress and trophoblast proliferation are associated with a whirlwind of expressional changes [139,140]. For example, levels of C19MC members significantly increase from 1st to 3rd trimester, while the opposite rings true for the maternally-imprinted cluster on chromosome 14 [110]. Such changes may be reflected in circulation, as reinforced by Pan *et al*. [141] who detected differential expression of circulatory miRs before and after parturition. This highlights the importance of timing when collecting samples, and the impact of circulatory miRs which can be detected practically at timeframes prior to symptomatic onset and treatment.

Across screening studies which profiled circulatory miRs, only Ura *et al*. [48] and Akehurst *et al*. [142] included samples collected before the 3^rd^ trimester, in plasma and serum samples respectively, but with no overlap in results. While plasma and serum results are comparable, serum samples have been noted to contain a higher level of miRs, suggested to be due to additional release of RNA from red blood cells during the coagulation process [143]. The placenta is one of the most abundant sources of exosomal miRs, and many PE-associated miRs found in placenta such as miR-210, -223, -181a, -126 and C19MC members, have also been observed to be differentially expressed in circulation [28] (S1 File). This could indicate roles in cell-cell (e.g. maternal-fetal) communication, or be merely a consequence of increased debris/apoptosis in cells in which they are highly expressed. In support of the former, there is evidence that miR-223 can be transferred via high-density lipoproteins to endothelial cells where it is thought not to be expressed, and from where it can target *ICAM-1* [144]. Corroborating miRs from results of antenatal circulatory profiling with those from tissue samples improves the capacity to define miRs with a potential long-term involvement in PE pathogenesis. Interestingly, some of the most frequently detected differentially expressed miRs across screening studies have also only been described in placental samples thus far. Examples include miR-584, and both miR-363 and -20 which are part of the miR-106a/363 cluster whose members appear to associate with PE severity. Jiang *et al*. [47] found miR-584 to target endothelial nitric oxide synthase (*eNOS*) and inhibit the migration of HTR8/SVneo trophoblast cells, although Western blot and Transwell insert assays showed the effect to only be slight with use of miR-584 mimics alone, in comparison to synergistic use with miR-355 which shares a target in *eNOS*. Studies using samples from early in gestation have great potential in revealing miRs which may only be transiently expressed, but with significance towards pathogenesis. This, in turn, could prove important for the development of miR biomarkers to assist with the prediction and diagnosis of PE.

The current predictive power of biomarkers for PE is limited. Viable biomarkers must be sensitive and specific enough to distinguish PE from other hypertensive conditions of pregnancy, at a GA early enough to facilitate clinical intervention, while accounting for multiple possible aetiological pathways. Given these requirements, a combination of biomarkers is likely to be optimal [145]. Angiogenic and anti-angiogenic factors hold some promise in this regard, with studies indicating their imbalance in maternal circulation to have predictive value for PE and its severity [22,146–148]. Indeed, recently, a quantified ratio of two serum angiogenic factors, sFlt-1 and PlGF, was measured to have a positive and negative predictive value of 36.7% and 99.3% respectively—an improvement upon traditional clinical parameters such as blood pressure [149]. However, changes in the levels of circulatory sFlt-1 appear to be mainly significant around 5 weeks prior to the onset of PE, and such changes may be more diagnostically sensitive in women who later develop eoPE [148,150,151]. Moreover, impaired angiogenesis alone is not pathognomonic of PE. Biomarkers are therefore unlikely to be prognostic in all cases—although they may highlight some of the most severe [152]. It is among these reasons that circulatory miRs may be of benefit. In the context of the suspected heterogeneity of PE, miR biomarkers have the potential to reflect a wider range of indicted pathways, including those involved in both eoPE and loPE, at more initial stages than is possible using angiogenic markers. Evidence already exists that serum miR-210 expression can identify pregnant women at risk for a hypertensive condition, including PE, at around 8–12 weeks prior to clinical onset [62]. Use of such a marker would ideally need to be in conjunction with other potential PE-specific miR biomarkers within panels, before being tested for predictive capability in a large and diverse prospective cohort. To that end, preliminary research using miR panels with samples taken during the 1^st^ trimester have suggested the viability of this method [153,154]. The future feasibility of miRs in this clinically diagnostic context is contingent upon further study of their altered expression at antenatal periods in PE individuals, as well as greater reproducibility in results between such studies, taking into account the various factors discussed in this review.

In examining differences in sampling methodology which could account for variability in results, crucial aspects include the timing and mode of delivery, as well as the sampling technique. Placental sample collection is a complex matter; collecting, processing and storage of samples all require attention to maximize a sample’s representation of the *in vivo* state. The timing of cord clamping can result in reduced placental weight, while labour-induced stress (ischemia-reperfusion and compression) can result in increased activity of the free radical generating xanthine oxidase, in addition to gene expression changes [155]. Most studies opted to match cases and controls for mode of delivery, while preferentially including individuals who delivered by caesarean. This is a useful near-constant for comparing studies. In addition to avoiding expression changes induced by labour and its associated stresses, matching cases with elective caesareans in controls avoids over-representing non-PE induced caesarean deliveries in control individuals.

One of the most significant sources of bias is in the sampling region. An oxygen gradient is purported to exist from the central placental region to the periphery, where the periphery also composes a larger portion of the total volume [155]. Therefore whole-tissue, or global sampling, must take into account representative volume and oxygenation differences across the placenta. Many studies omit details of sampled placental region, leaving the assumption of global sampling. However, global sampling may neglect region-specific variation in miR expression, masking differential expression in placental regions which may otherwise contribute towards pathogenesis. Wyatt *et al*. [156] found many hypoxia-related transcripts to have a higher expression in the lateral-chorionic regions than in medial regions, including the hypoxia-induced VEGF [157]. Differences in perfusability from medial to lateral regions across the placenta are likely at least partially responsible for expression changes, as has been shown for certain anti-oxidant enzymes [158]. Importantly, regional variability has also been substantiated for miRs in candidate studies which involved investigating expression specifically from the chorionic and basal plates of the placenta (Table 4).

**Table 4:** List of differentially expressed miRs found in chorionic and/or basal plate regions of PE placentas.

Chorionic Plate (Fetal)	Both Plates	Basal Plate (Maternal)	Reference
No significant change	*N/A*	*125b-1-3p*	[159]
No significant change	*N/A*	*210*	[63]
195, 18a, 379, 519e-5p, 17, 19b, 92a	223, 218, 411	*210*, *30a-3p*, *518b*, *524*, *17-3p*, *151*, *193b*, *214*	[41]
18b, 19b	*20b*, 92a, 363	*106a*	[50]

All studies included used qRT-PCR for determining expression levels. Samples were collected from a central placental location. Upregulated miRs are given in *italics*. Downregulated miRs are not italicized.

Spatial patterns of miR expression represent a key consideration when examining contributions to pathogenesis. Interestingly, there appears to be a clear dichotomy in direction of change of differentially expressed miRs between chorionic and basal plates in PE, perhaps reflecting changes in vasculature and oxygenation across placental regions. Localization of miR-210 has previously been shown in the basal plate interstitial trophoblasts and in the syncytiotrophoblasts of the chorionic villi, which sprout towards the basal plate—reinforcing studies of its observed differential expression in basal plate samples [46,70]. Overall, regional expression differences highlighted here, including directions of change, correlate strongly with those of hypoxia-regulated miRs from studies in cancer [160]. All included studies collected samples from medial placental locations, near the umbilical insertion point. As is the case with hypoxia-regulated transcripts such as connective tissue growth factor (*CTGF*) under normal physiological conditions, it is expected that hypoxia-regulated miRs increase in expression from medial to lateral regions—particularly in the chorionic plate. Given that CTFG has been shown to increase VEGF-dependent angiogenesis via miR-210, it would be interesting to see if any overlap in regional expression exists in PE [161].

The timing and storage of collected samples poses an addition layer of methodological challenge which could account for variability. Bravo *et al*. [162] emphasised the inherent instability of miRs, demonstrating rapid degradation of samples stored at -80c for 3 days in a manner which differed according to the specific miR and was seemingly unrelated to both isolation method and relative abundance of the miR. Similarly, an investigation into plasma storage of extracellular miRs found significant reductions in levels of particular miRs (including C19MC members) after long-term storage of just over a year, with samples stored for < 2 months having the least effect on degradation [163]. Differences in storage duration of used plasma samples could therefore have introduced bias into results from circulatory profiling studies. In the included studies, the majority of placental samples were collected immediately after deliver and either processed soon after, or snap-frozen at -80c for later processing. Two studies however, by Choi *et al*. [37] and Noack *et al*. [129], profiled expression in formalin-fixed, paraffin-embedded (FFPE) tissues. Both FFPE and frozen tissue samples are well-matched for making comparisons, but FFPE tissue samples, as compared to cells, may harbour differences in miR expression levels related to sample preparation [164,165]. It is thought that this may be due to latent RNAse cleaving of precursor miRs in FFPE samples kept at room temperature.

### Isolation and profiling

**Fig 7:**
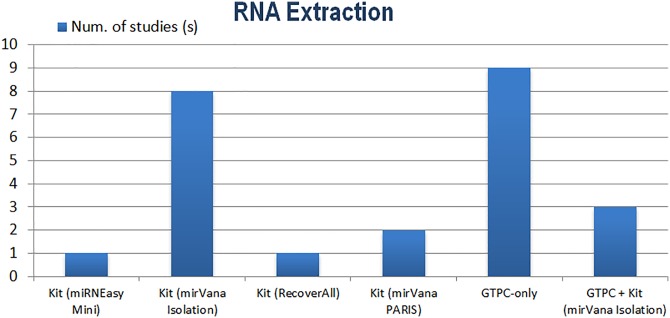
RNA extraction and isolation methods used across all included screening studies.

Prior to profiling, RNA extraction and isolation methods can significantly influence determinations by defining the set of miRs available for discrete hybridization, amplification or sequencing [166,167]. The majority of screening studies included in this review used isolation methods based on either Guanidinium thiocyanate-phenol-chloroform (GTPC—e.g. TRIzol) only, which isolates total RNA, or column-enrichment kits, which can enrich small RNAs (Fig 7).

Podolska *et al*. [168] demonstrated that different RNA isolation techniques (GTPC or column-based) can result in distinctive microarray expression clusters, with some miRs appearing particularly sensitive to bias in isolation procedure—including small nuclear RNAs typically used as controls. RNA isolation kits have also been shown to differ in yield, purity and consistency of captured RNAs [169]. While isolation methods were not all consistent across studies, it can be considered a strength that most studies opted to use either TRIzol or mirVana. Of these, the latter may be more useful for NGS profiling with respect to the identification of miRs and reproducibility [170].

The most utilised approaches for miR profiling are microarrays, qRT-PCR and NGS—each bearing advantages and caveats which can have profound effects on results. Microarrays are the most frequently employed across screening studies. Probe design for microarrays is a delicate matter; probes must differentiate between precursor and mature miRs, highly-related miRs (potentially differing by a single nucleotide) and account for differences in thermo-stability between miRs. Considerable inter-platform differences between microarrays have been noted, and the lack of consensus in microarray platform across screening studies may account for observed variability [171,172]. Kuo *et al*. [173], in a comparison of all commercial and custom platforms, found less concordant results between different platforms, than between different labs using the same platform. Differences in specificity and sensitivity of platforms (e.g. probe design), platform-specific stringency criteria for detecting calls and downstream data analysis are all likely to introduce bias [174]. The lack of sensitivity in microarrays may overlook miRs which display inconsistent hybridization or amplification [175]. These factors highlight the importance of rigorous and standardized approaches to microarray profiling, appropriate normalization procedures and independent validation by NGS or qRT-PCR.

**Fig 8:**
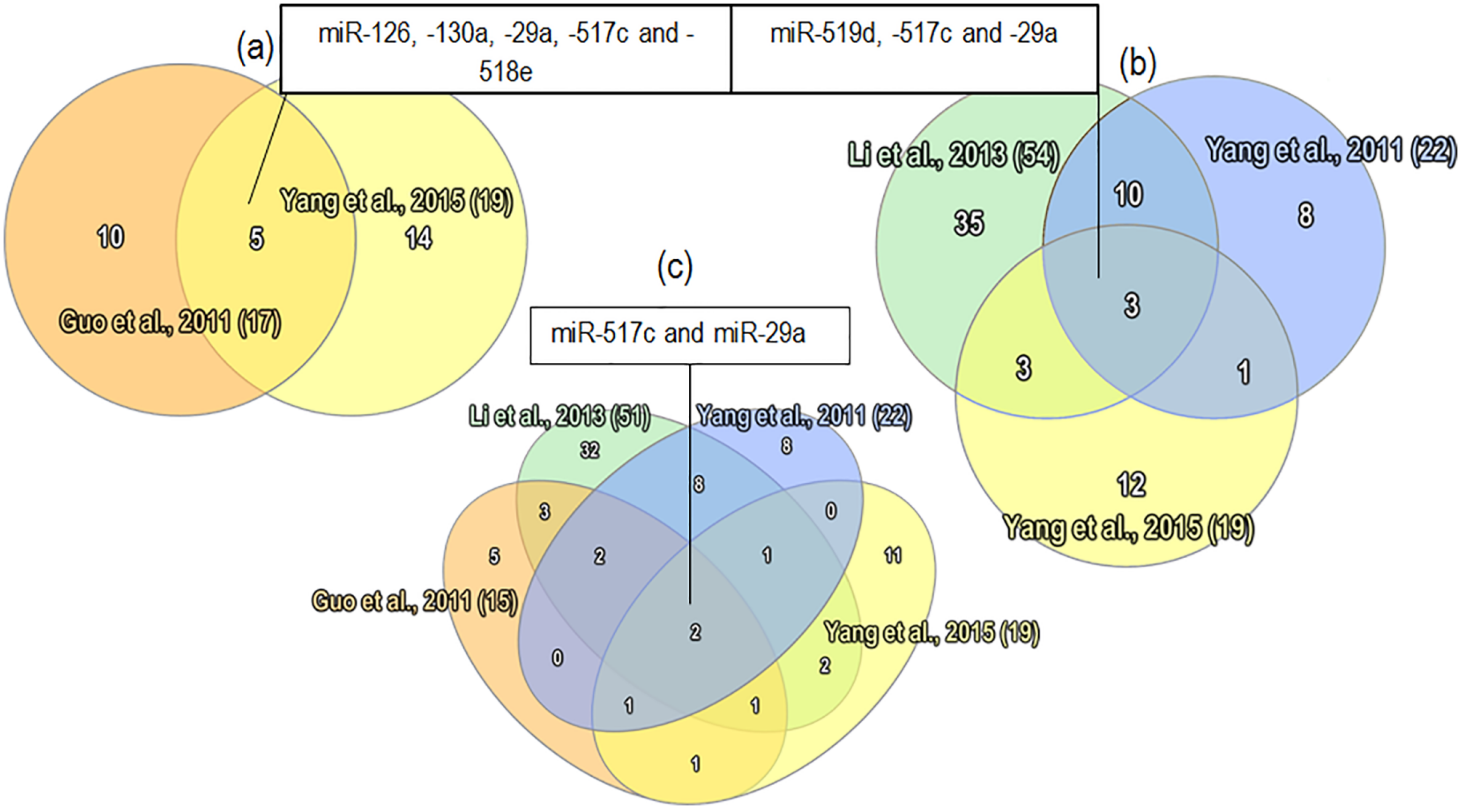
Overlapping results from SOLiD-based NGS screening studies. a) Profiling in placental samples with similar methodologies b) Profiling in circulatory (plasma/serum) samples c) Profiling across all sample types.

The second most prolific profiling method is NGS, considered the new “gold standard” technique in large-scale studies. The advantages of NGS compared to array-based platforms include a determination of absolute miR quantity, uncovering novel miRs without a reliance on pre-defined capture probes and detection of miR isoforms, or isomiRs [176]. For example, Guo *et al*. [38] was able to conduct a study on isomiR expression between PE and control individuals, noting a greater representation of isomiRs with 3’additions in control samples as compared to PE cases, where such additions could alter the stability and binding strength of miRs. Most screening studies utilising NGS opted for SOLiD sequencing. In an investigation of these, overlap existed in all SOLID-based studies profiling both placental and circulatory samples for miR-29a and -517c (Fig 8c).

Interestingly, many of the overlapping miRs from SOLiD-based studies appear to be almost exclusively from NGS-based studies. This perhaps reflects the sensitivity of this technology in the detection of particular miRs, and is indicative of the comparability in results between SOLiD-based studies. An additional candidate study utilising SOLiD sequencing by Pan *et al*. [141] also detected differential expression of miR-29a and showed this to be significantly downregulated after parturition in control cases. When Illumina-based studies were included in the overlap investigation, miR-223 was among the most prevalent miRs detected in NGS studies (4/6) and consistent representation of C19MC members in PE cases occurred in 5/6 NGS-based studies. A high correlation between SOLiD and Illumina sequencing technologies has been demonstrated, as well as good comparability between SOLiD and qRT-PCR results—further illustrating the benefits of NGS-based profiling methods [177].

Lastly, qRT-PCR has been considered the “gold standard” of small-scale miR measurement due to its accuracy, sensitivity and specificity. Hence, qRT-PCR is typically used to validate microarray or NGS results. Good intra-platform comparability reportedly exists between microarray and qRT-PCR results, with variability in FC values attributed to differences in cDNA preparation or data normalization [171,175]. By contrast, array-based qRT-PCR results have been noted to have a low correlation with microarrays, with the effect more prominent in miRs with lower expression [178]. The sensitivity and low sample requirement of qRT-PCR is particularly advantageous for evaluating circulating miR profiles, where levels of miRs may be exceedingly low [179]. A subset of screening studies used qRT-PCR for profiling, often with arrays (e.g. OpenArray) which have been demonstrated to be superior to traditional microarray-based profiling [178]. However, interplatform differences have been shown to exist between different qRT-PCR platforms when addressing the differential expression of miRs, which could account for variability across qRT-PCR-based studies [180].

### Data normalization

Normalization is an imperative step to eradicating technical bias not attributable to genuine biological difference—for example, with RNA sampling and input. Lack of normalization with microarrays has been shown to result in low conformity with qRT-PCR results [181]. For microarray-based studies, no assumption can be made of uniformity in total miR content per cell, nor of the quantity of miRs relative to total RNA, creating a challenge for standard normalization methods. Of the few microarray studies which included details of normalization, aside from typical methods of calculating mean spot intensities with or without subtracting the median background, other methods involved relative adjustment to average intensity of a control miR (either added or endogenous), global median centring and locally weighted scatterplot smoothing (LOESS). In a comparison of miR microarray normalization methods, Hua *et al*. [181] noted that results normalized using LOESS had the highest correlation with qRT-PCR results, though it is unlikely that this method will be suitable for all purposes—thus, the method must fit the purpose.

**Fig 9:**
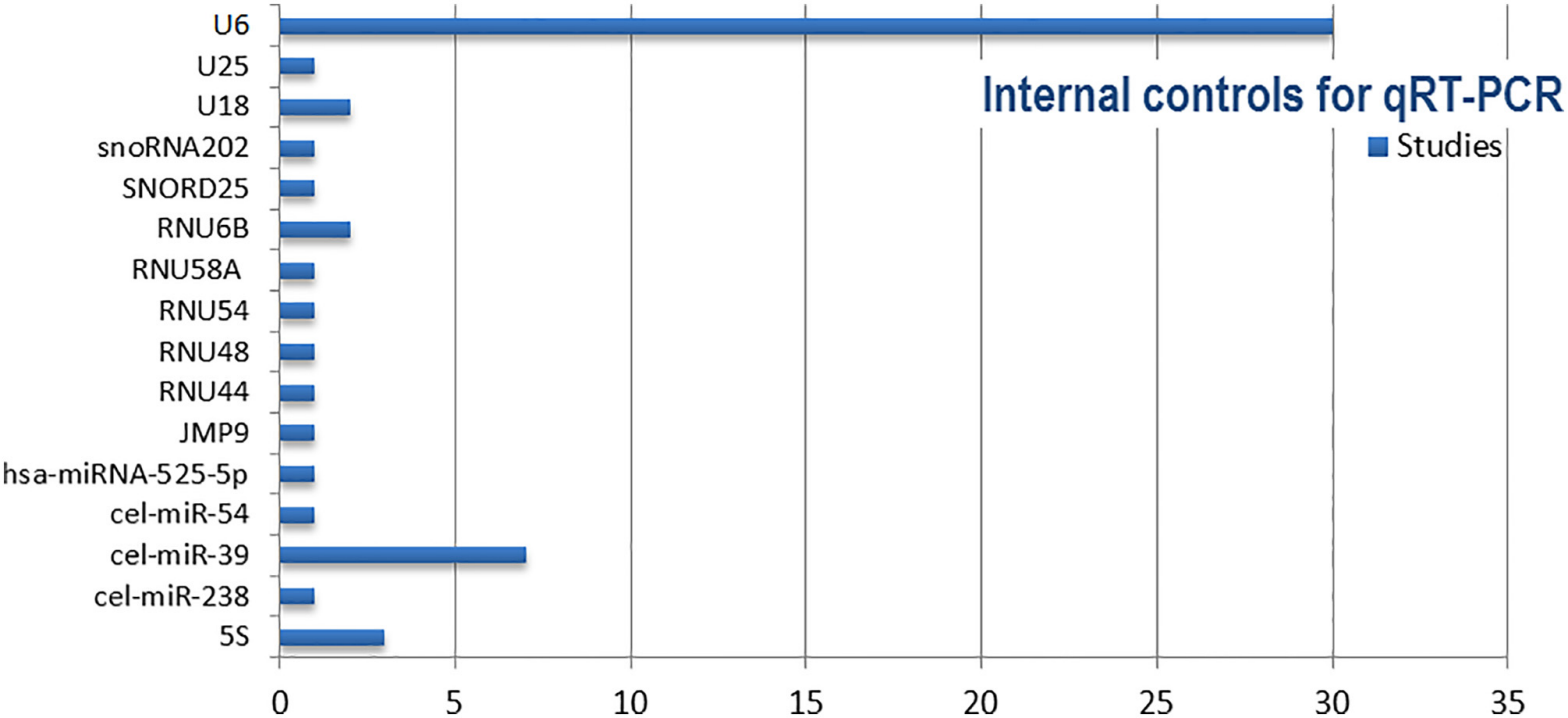
Internal controls used for qRT-PCR (both array-based and stem-loop) across screening and candidate studies mainly consisted of the small nuclear RNA, U6.

Most qRT-PCR-based studies used small RNAs as internal controls for normalization: either spiked-in cel-miR-39 (for circulatory determination) or the small nuclear RNA, U6 (Fig 9).

Conformity of internal controls can be considered a positive when collating results. However, most studies did not state prior validation of internal controls to ensure constant expression and suitability before use. The most frequently used endogenous control, U6, is processed differently to miRs, meaning that expression could be altered under different experimental conditions [176]. Presumptions of constant expression of well-used controls in all samples under different experimental conditions can therefore lead to invalidating results. Alternative normalization methods include global mean normalization, a method based on the mean expression level of all detected miRs, which may be more suitable than use of endogenous controls for profiling a large set of unbiased miRs in a genome-wide manner [182].

Where normalization of NGS data is concerned, both LOESS and quantile-based normalization have been indicated to correlate well with qRT-PCR results [183]. A more recently proposed, and perhaps simpler approach termed RUV (removed unwanted variation) was also able to provide robust estimates in RNA-seq differential expression analyses, in some cases outperforming cyclic LOESS and upper-quartile strategies, particularly when spiked-in RNA controls were used [184]. However, as with normalization in general, any assumptions made by the chosen method must be appropriate for the data under scrutiny.

Lastly, although FC is frequently used to quantify the differential expression of miRs, values constituting meaningful change *in vivo* are unclear. Threshold values for FC significance varied between studies; significant upregulation was generally considered to be a FC >2 or >1.5, with few differentially expressed miRs showing a FC >3. However, even a large FC value for a miR expressed at low levels does not confer any meaning for functional significance. This represents an area of considerable uncertainty when comparing results and a weakness of this review is in the inclusion of results proposed significant by each study, as determining an arbitrary global significance threshold for FC would require access to raw data.

## Conclusions

PE appears to be a heterogeneous condition within which many pathogenetic routes converging on similar phenotypes have been implicated—permitting it well deserving of its often-claimed title as the “disease of theories”. Since the initial study delving into miR expression in PE by Pineles *et al*. [30], a wave of studies examining miR expression in PE have steadily emerged and the tide of data risen ever higher with no clear consensus on which, and how, miRs may associate with pathogenesis.

In spite of this, and methodological variation among studies, certain miRs have emerged repeatedly and appear to associate strongly with PE. Many elucidated in this review, such as miR-210, miR-223 and miR-126/126*, impinge on a spectrum of diseases. Through candidate studies each can be intertwined with some proposed aspect of PE pathogenesis—as with hypoxia and the HIF-1α/miR-210/ISCU pathway towards mitochondrial abnormalities. Other PE-associated miRs segregate into distinct families with predominately placental expression patterns, such as the prolific miR-515 family clustered within the expansive, primate-specific C19MC. Epigenetic alterations may hold the key to aberrant expression of this cluster. This paternally-imprinted cluster, whose members appear prevalent in secreted exosomes, could be a means by which the fetus modulates adverse maternal immune responses. However, many C19MC miRs, such as miR-518b, still lack verified target genes outside of cancer-based studies, despite frequent association with PE. Further investigation is therefore essential in order to understand their precise contributions. By expanding the question of contribution towards PE classification, certain miRs appear to correlate with PE severity—as with miR-210, -223 and -181a. Some miR families and clusters may also be closely tied to more severe forms of PE, such as the miR-106a/363 cluster and miR-15 precursor family from which the prevalent miR-195 and -16 emerge. Patterns of miR expression could help link similar pathological processes to particular classifications of PE, resolving ambiguity, for example, between eoPE and sPE cases. Exploring these questions further may therefore prove useful in disentangling the web of PE definitions through strongly associating miRs linked to pathogenic pathways (e.g. IL-6/miR-223/STAT3) and beyond this, for ascertaining the clinical value of miRs.

PE-associated miRs have the potential to serve as viable biomarkers of the condition. This could be through the creation of miR-based panels with the capacity to aid in diagnosis, heralding the onset and/or severity of PE. In a therapeutic context, transiently modulating the expression of genes implicated in pathways relating to pathogenesis using miR mimics and inhibitors may also have clinical value, as with miR-126 mimics and the angiogenesis-related PIK3R2/PI3K/Akt pathway. However, a current lack of consensus across studies with regards to PE-associated miRs limits their capacity to be of wider, practical clinical value. Ultimately, any clinical value is predicated upon resolving current methodological challenges which may account for variability in results, allowing for a greater degree of standardization among studies. Challenges include the definition and classification of PE used, sampling technique, RNA extraction method and crucially, the chosen platform for miR profiling. Furthermore, accurate PE animal models will continue to prove important for allowing more rigorous determinations of miR functionality in the context of entire biological systems.

Overall, this systematic review touches upon a promising and expanding area of research, propelled by new technologies, such as NGS, which are better able to clarify the pictures formed by prior studies, and driven by the ever-present threat of the condition. It is hoped that further exploration of miR expression in PE, including a systems-level approach to unearthing target genes and pathways, will continue to inform domains which may be of relevance to pathogenesis and advance our present understanding of PE.

## Supporting Information

S1 File Supplementary data file containing all included differentially expressed miRs across studies. (XLSM)

S2 File PRISMA 2009 Checklist. (DOC)

S3 File PRISMA 2009 Flow diagram. (DOC)

## Abbreviations

PE: Pre-eclampsia / Pre-eclamptic
eoPE / loPE: Early-onset pre-eclampsia / Late-onset pre-eclampsia
mPE / sPE: Mild pre-eclampsia / Severe pre-eclampsia
miR: microRNA
FC: Fold-change
GA: Gestational age
IUGR: Intra-uterine growth restriction
SGA: Small-for-gestational age
qRT-PCR: Quantitative reverse transcription PCR
NGS: Next-generation sequencing
HIF-1α: Hypoxia-inducible factor 1α
TLR: Toll-like receptor
STAT: Signal transducer and activator of transcription
FOX: Forkhead box protein
PIK3R2: Phosphoinositide-3-kinase regulatory subunit 2
PI3K: Phosphatidylinositide 3-kinase
C19MC: Chromosome 19 microRNA cluster
