# Supplementary material for: Systematic Review of Micro-RNA Expression in Pre-Eclampsia Identifies a Number of Common Pathways Associated with the Disease
Source: PLoS One. 2016 Aug 16;11(8):e0160808. doi: 10.1371/journal.pone.0160808 (PMC4986940; doi:10.1371/journal.pone.0160808)
Supplement: S3 File — (DOC) [file pone.0160808.s003.doc]

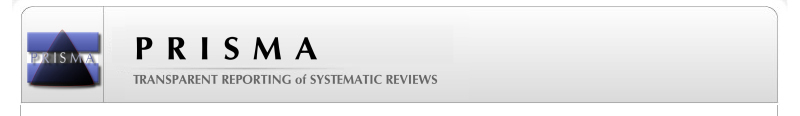
**PRISMA 2009 Flow Diagram**

**Screening**

**Included**

**Eligibility**

**Identification**

Records identified through database searching
(n = 119 )

Additional records identified through other sources
(n = 125 )

Records after duplicates removed
(n = 177 )

Records screened
(n = 177 )

Records excluded
(n =87 )

Full-text articles assessed for eligibility
(n = 90 )

Full-text articles excluded, with reasons
(n = 32 )

Studies included in qualitative synthesis
(n = 58 )

Studies included in quantitative synthesis (meta-analysis)
(n = 58 )
